# Placental hypoxia, endoplasmic reticulum stress and maternal endothelial sensitisation by sFLT1 in pre-eclampsia

**DOI:** 10.1016/j.jri.2015.07.004

**Published:** 2016-04

**Authors:** D. Stephen Charnock-Jones

**Affiliations:** Department of Obstetrics and Gynaecology & NIHR Biomedical Research Centre, University of Cambridge, The Rosie Hospital, Robinson Way, Cambridge CB2 0SW, UK

**Keywords:** Hypoxia, ER stress, Unfolded protein response, sFLT1, Pre-eclampsia

## Abstract

The human placenta is a multifunctional organ that grows and adapts to increasing fetal demand and fluctuations in the intrauterine environment. It is subjected to physiological and pathological changes in local oxygenation, both of which induce adaptive changes. In early pregnancy a low PO_2_ is the normal physiological state and this is not hypoxic—there is no perturbation of ATP/ADP ratios and, if the placenta is sampled very rapidly, little HIF1α is detected in human first-trimester placental villi. Nonetheless, HIF1α can be increased and activated by culture. However, the placenta does show evidence of stress under pathological conditions. For example, in cases of pre-eclampsia where delivery by caesarean section is necessitated for maternal well-being before 34 weeks’ gestation, placental endoplasmic reticulum stress is evident. Cases delivered ≥34 weeks are indistinguishable from normal term controls. One consequence of placental stress, whether oxidative, related to the endoplasmic reticulum or immunological, is that factors are released into the maternal circulation, which affects the endothelium, leading to the maternal syndrome. Soluble FLT1 may contribute directly to this and the most likely mechanism is direct action on the maternal endothelium. sFLT1 is able to form a heterodimer with cell surface VEGF receptors and is therefore able to have a dominant negative effect (in addition to acting as a competitive inhibitor by simply binding vascular endothelial growth factor A [VEGFA] and placental growth factor [PlGF]). This leads *in vitro* to the sensitisation of endothelial cells to low levels of TNFα.

## Highlights

• The concept of “placental hypoxia”.
• Placental endoplasmic reticulum stress and maternal sensitivity to pre-eclampsia.
• How soluble FLT1 sensitises maternal endothelium to inflammatory mediators *in vitro*.

## 1 Placental stress

In human pregnancy the outermost layer of the placental villi is formed of the syncytiotrophoblast. This is a continuous barrier and mediates exchange functions between the fetal and the maternal circulations. It also performs an important endocrine function, secreting numerous proteins. Many of the proteins necessary for these two functions are glycosylated. Some of the most abundantly secreted placental proteins are in fact the pregnancy-specific glycoproteins and, in ruminants, pregnancy-associated glycoproteins (PAGs) (Moore and Dveksler, 2014, Wallace et al., 2015). In addition, the well-known placental glycoprotein hormone family in humans includes placental lactogen and hCG (Fournier et al., 2015). It is also worth noting that protein synthesis, such as is necessary to produce large amounts of secreted glycoprotein hormones, consumes considerable amounts of cellular energy; indeed, the formation of a single peptide bond consumes a total of four high-energy phosphate groups.

## 2 Placental hypoxia?

A considerable portion of the current literature relating to pre-eclampsia implicates some form of “stress”, which affects the placenta, leading to compromise of its function. This in some way leads to the release of factors that subsequently affect maternal and fetal physiology. Much of the focus of the placental stress is attributed to a defect in spiral arterial remodelling that in turn may be due to perturbation of immunological interactions with decidua (Burton et al., 2009). The “stress” that the placenta experiences has been variously categorised as oxidative stress, endoplasmic reticulum stress, and immunological stress (Roberts and Bell, 2013). Particularly in relation to oxidative stress, many papers describe the placenta as “hypoxic” during the first trimester. Indeed, such terminology is commonly used when describing normal placental development and is based on the now well-accepted data that the PO_2_ in the first trimester of the human placenta is approximately 20 mmHg (Jauniaux et al., 2000). However, the authors who made the primary observations do not describe placenta as “hypoxic” anywhere in the manuscript. They carefully report the PO_2_ and describe placental development as normally occurring in a low oxygen environment. Thus, this is the normal physiological state and there are no significant differences in the ATP/ADP ratio nor in the concentrations of NAD^+^, lactate or glucose among first-, second- or third-trimester samples (Cindrova-Davies et al., 2014). Indeed, the local PO_2_ is different in different tissues; for example, in liver and muscle it is ∼30 mmHg, in the kidney it is ∼50 and 10–20 mmHg in the cortex and medullar respectively and in the superficial regions of the skin it is ∼8 mmHg (Carreau et al., 2011). Recent work has shown that there is considerable variation in oxygen tension over short distances within the bone marrow (Spencer et al., 2014). Thus, the PO_2_ of the normal first-trimester placenta is not so different from many of these values.

Many of the key cellular responses to low oxygen are mediated by hypoxia-inducible factor 1 and several authors have reported the presence of this protein (or more accurately the α subunit) in first-trimester villi (Ietta et al., 2006). This has frequently been cited as evidence that the first trimester villi are “hypoxic”. However, HIF1α, while it is clearly induced by low oxygen, can also be induced by a variety of other stresses that include oxidative stress. Furthermore, the induction, or more accurately the stabilisation of the HIF1α, can also be influenced by a variety of stress response kinases. Thus, inferring that a tissue is hypoxic merely on the basis that the HIF1α is detected may be imprudent (Pringle et al., 2010, Kuschel et al., 2012). We have recently shown that first-trimester human villi collected using a method akin to chorionic villi sampling in which the tissue is removed and frozen within seconds contain only modest amounts of HIF1α. If such tissue is cultured then the level of HIF1α immunostaining rises dramatically. The level of this staining is affected neither by the oxygen level in culture nor the presence of hydrogen peroxide, indicating that the simple level of oxygen is inadequate to explain the increase. Furthermore, samples of placental villi collected by more conventional curettage-based methods show strong staining for HIF1α. These data indicate that the level of HIF1α is extremely sensitive to the post-collection environment, but when the interval between collection and freezing is minimised, the levels are low. This is likely to reflect the *in vivo* state. The HIF1α that is detected by immunostaining is functionally active as the level of vascular endothelial growth factor A (VEGFA) mRNA rises and placental growth factor (PlGF) mRNA decreases upon culture of the villi. This response is independent of oxygen and the response (in terms of VEGFA mRNA induction) can be largely blocked by inhibiting the action of the p38 stress kinase (Cindrova-Davies et al., 2014).

Thus, while the placenta in pre-eclampsia may well experience a variety of forms of stress it is inaccurate to say that the normal state of the first-trimester villi in human pregnancy is “hypoxic”.

## 3 Endoplasmic reticulum stress

Endoplasmic reticulum stress may be more accurately described as the unfolded proteins response (UPR), or the unified stress response. These different names reflect the fact that multiple different stresses induce similar changes and that much of the response is dependent on the presence of unfolded proteins present in the endoplasmic reticulum. Proteins that are to be secreted or glycosylated are translated by the ribosomes on the rough endoplasmic reticulum and translocated into the lumen of the ER. Here, they are in an environment that facilitates correct three-dimensional folding (owing to the appropriate ionic environment, pH and the presence of chaperones and protein disulphide isomerases). They are also partially glycosylated, a process that continues in the Golgi apparatus. These processes are homeostatically regulated and the cell is able to sense the presence of unfolded proteins and modulate its metabolism accordingly. For example, under conditions of elevated ER stress there is an increase in transcription of a specific subset of genes that lead to an increase in the ER protein folding capacity (Yamamoto et al., 2007). PERK and IRE1 mediate translational control; in the case of PERK, by phosphorylating eIF2α to suppress translation and IRE1 induces mRNA decay, also reducing the translation load and hence the necessity for folding capacity within the ER (Oakes and Papa, 2015, Walter and Ron, 2011).

Specific phosphorylation of serine 51 in eIF2α is a rapidly induced switch to control translation–phosphorylation inhibiting cap-dependent translation. Mutation of this serine (such that eIF2α cannot be phosphorylated and therefore cannot be inactivated by PERK or other kinases) leads to profound ER stress (Scheuner et al., 2001). We have shown previously that mice bearing this mutation show marked ER stress in the junctional zone of the placenta, whereas the labyrinth (the transfer region) of the placenta is relatively unaffected (Yung et al., 2012). The differentiation of the various placental cells and the placenta is also altered. The *in vitro* differentiation of trophoblast stem cells can also be modulated by conditioned media obtained from mutant or wild type fibroblasts. This indicates that the factors produced by these fibroblasts are sensitive to ER stress and that their functions can be perturbed in such a way that the normal course of trophoblast differentiation is altered (Yung et al., 2012).

However, while this is clear in the mouse, it is not possible to use similar methods in the study of humans. Nonetheless, we have evidence that ER stress is a feature of pre-eclampsia and the severity of this may also shed light on maternal sensitivity to this condition (Yung et al., 2014).

**Fig. 1:**
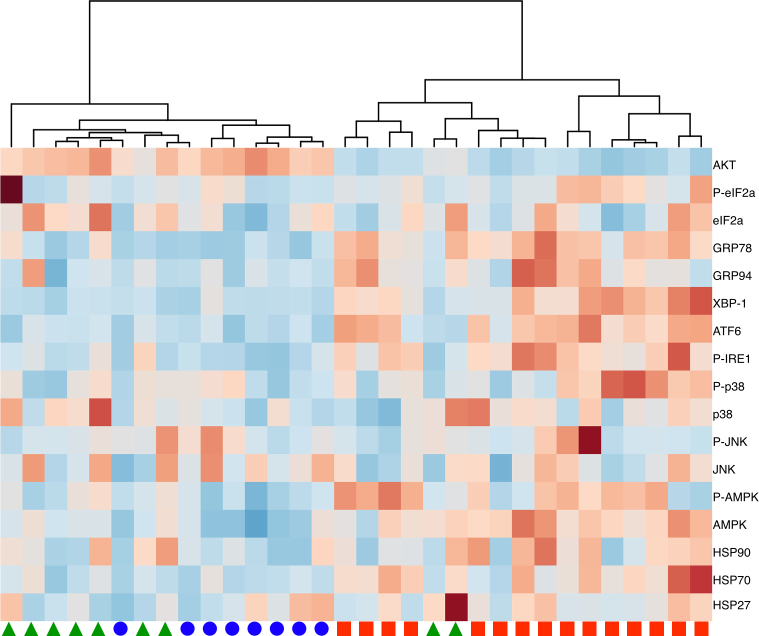
Hierarchical clustering of placentas based on the analysis of ER stress response and signalling intermediates. Red squares represent placenta delivered for clinical necessity before 34 weeks’ gestation, green triangles indicate placentas delivered after 34 weeks’ gestation and blue circles represent normal term caesarean controls.

We evaluated the phosphorylation level of many of the effectors and mediators of the UPR and several of the kinases activated in the stress response pathway. We have tested samples of placenta collected from either normotensive term controls or from patients with pre-eclampsia who were delivered by caesarean section. The requirement for caesarean delivery was used as the indicator of the severity of maternal disease and the gestational age at the time of caesarean noted. The samples are defined by the gestational age at caesarean, and not at the time of the onset of disease. Hierarchical clustering of these data reveals that all the placentas delivered owing to clinical necessity at <34 weeks cluster in one branch and all but 2 of those delivered ≥34 weeks and all the normotensive controls cluster in the other major branch; indicating the similarity among placentas in the latter two groups (Fig. 1) (Yung et al., 2014). These findings support the concept that cases of early-onset pre-eclampsia are predominantly due to placental abnormalities, while those of late-onset pre-eclampsia are likely the result of increased maternal sensitivity to the pro-inflammatory environment of pregnancy, because of metabolic or other disturbances (Yung et al., 2014).

## 4 Dominant negative action of sFLT1, sensitising endothelial cells

One consequence of the placental “stress” whether oxidative, endoplasmic reticulum or immunological is that factors are released into maternal circulation which affect the internal endothelium leading to the maternal syndrome. There has been considerable interest in the possible role of PlGF and soluble FLT1 and these factors (along with others) are being evaluated as possible biomarkers (predictive) of pre-eclampsia (Benton et al., 2011, Rana et al., 2014). It has also been suggested that sFLT1 might directly contribute to maternal endothelial dysfunction (Maynard et al., 2003, Powe et al., 2011). However, the presence of these factors and soluble endoglin has been more recently described as “the anti-angiogenic state”, although this phrase does presuppose that angiogenesis occurs during pregnancy and that these factors in some way interfere with this. Clearly, angiogenesis does occur in the placenta, but once placentation is complete there is little angiogenesis in the decidua. A distinction needs to be made between angiogenesis (the growth of new blood vessels from pre-existing vessels) and vascular remodelling, which clearly occurs in the decidual spiral arteries. Nonetheless, even if there is little maternal angiogenesis, it is highly likely that the presence of these factors in the maternal circulation can have a direct effect on the maternal endothelium.

**Fig. 2:**
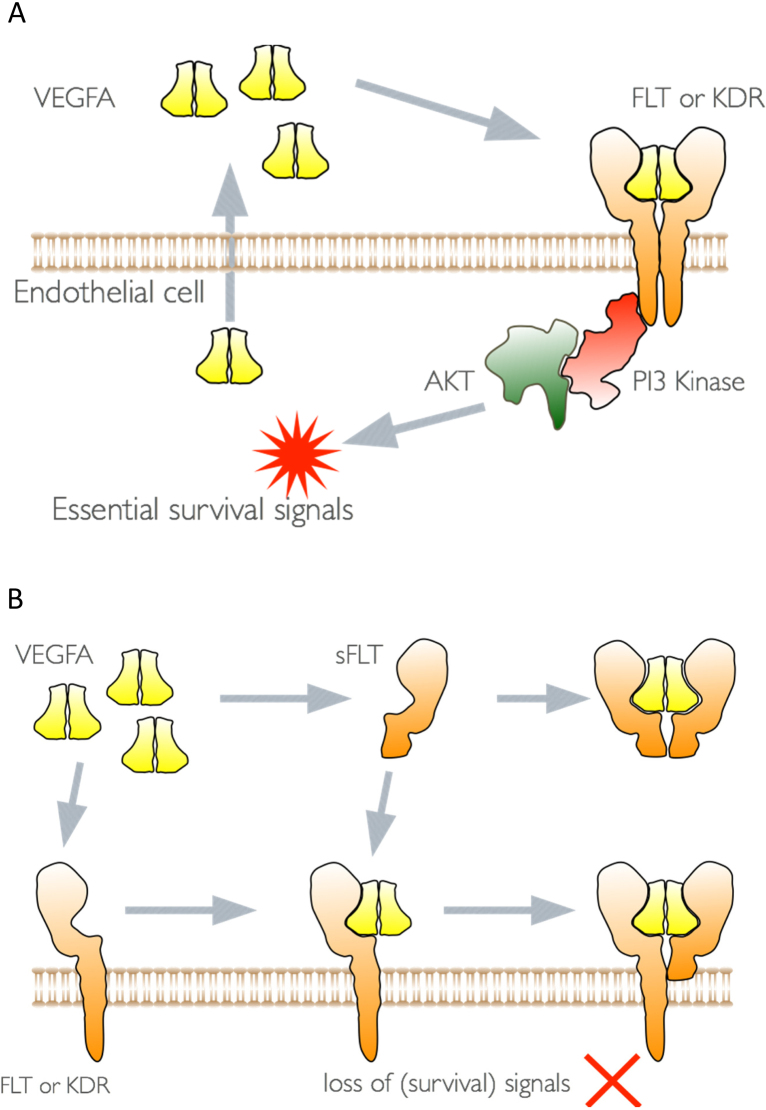
Schematic of (A) the autocrine action of VEGFA on endothelial cells, and (B), the dominant negative actions of sFLT1.

Vascular endothelial growth factor A (VEGFA) is widely recognised as a potent angiogenic factor that is produced by numerous different cell types and acts directly on endothelial cells to promote vascular permeability, endothelial proliferation and migration (Ferrara et al., 2003). It is also a heparin-binding growth factor and is therefore capable of being bound and sequestered by extracellular matrix and heparan sulphate proteoglycans from which it can be released by the action of proteases (Houck et al., 1992, Keyt et al., 1996). These biochemical properties for VEGFA complicate a simple interpretation of its actions. Further complication is caused by the fact that endothelial cells themselves produce VEGFA; indeed, they are dependent on it for survival (Lee et al., 2007). This is represented in cartoon form in Fig. 2 in which the VEGF homodimer is released by the endothelial cell and acts directly on one or other of the VEGF receptors. Heterodimers for the VEGF receptors are also present on endothelial cells and VEGFA may act through such heterodimers (Barleon et al., 1997, Cudmore et al., 2012). It is also possible that the actions take place entirely within the endothelial cell, a so-called “intracrine” effect (Liu et al., 2012). This has been shown as a mechanism by which VEGFA mediates the survival of immature haematopoietic cells (Gerber et al., 2002). It is well recognised that soluble FLT1 is a very effective inhibitor of VEGF as it binds to VEGFA with high affinity and effectively competes with the cell surface membrane-bound receptors for free VEGFA (Kendall and Thomas, 1993). However, the soluble receptor is also able to form heterodimer with the cell surface receptors and thereby inhibit any signalling action that they may have. This mechanism of action of soluble FLT1 is frequently overlooked and interaction with the endothelial cells in this manner has the potential to subtly alter endothelial cell behaviour over a long time period.

We have previously shown that pre-incubation of endothelial cells with soluble FLT1 profoundly alters the sensitivity of these endothelial cells to low doses of TNFα (Cindrova-Davies et al., 2011). This is reflected in the binding of leucocytes to these cells, the level of ICAM, VCAM, Von Willebrand factor and endothelin. This sensitisation of the endothelial cells is dependent on VEGF receptor signalling, as the enhancement of sensitivity can be blocked by a tyrosine kinase inhibitor (SU5614) anti-KDR, anti-FLT1 antibodies and also siRNAs targeting KDR and FLT1. In each of these instances, the sensitivity to TNFα is increased, but this is not further enhanced by pre-treatment with soluble FLT1. These data implicate the endogenous VEGF receptors in cellular sensitivity to TNFα (Cindrova-Davies et al., 2011). These data provide a mechanism by which the circulating level of sFLT1 may influence multiple endothelial endpoints, many of which have been previously shown to be dysregulated in pre-eclampsia.

**Fig. 3:**
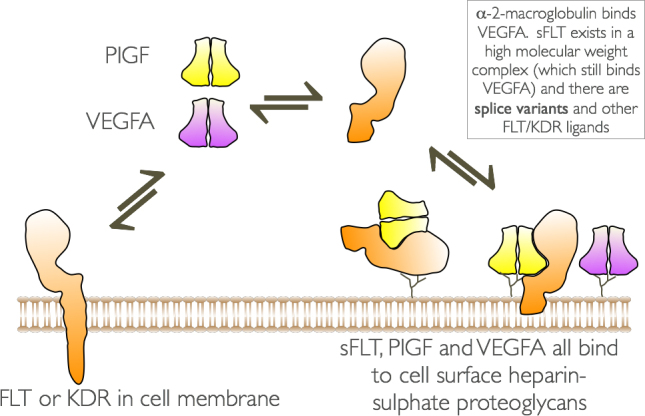
Summary of the complex interaction of some the VEGF family members, VEGF receptors and cell surface heparan sulphate proteoglycans.

The complex interactions between VEGF family members (particularly VEGFA and PlGF) the membrane-bound forms of the receptor, and the soluble receptor are all further complicated by the fact that each of these interacts with heparan sulphate proteoglycans on the cell surface and it is not clear how they affect the intracellular signals required for endothelial cell maintenance and quiescence (illustrated in Fig. 3).

## 5 Conclusion

The placenta is subject to a variety of stimuli to which it must adapt, with the level of oxygen receiving the most attention. The normal pO_2_ in the intervillous space in the first trimester of human pregnancy is low, but should not be described as hypoxic. sFLT1 is released from the placenta under a variety of “stress” conditions and is strongly implicated in the maternal syndrome of pre-eclampsia. It can directly affect endothelial cells by forming heterodimers with cell surface receptors and can, at least *in vitro*, alter the endothelial sensitivity to TNFα. However, the interactions among VEGF family members, cell-surface VEGF receptors and heparan sulphate proteoglycans are complex and the local effects hard to predict. Nonetheless, it is likely that this will be a factor contributing to variation in maternal sensitivity to pre-eclampsia.

## Financial support

This work was supported by the Wellcome Trust (84,804/2/08/Z) and the NIHR Cambridge Comprehensive Biomedical Research Centre.

## Disclosure

The author has nothing to disclose.
